# Immune profiles and clinical outcomes between sepsis patients with or without active cancer requiring admission to intensive care units

**DOI:** 10.1371/journal.pone.0179749

**Published:** 2017-07-10

**Authors:** Wen-Feng Fang, Yu-Mu Chen, Chiung-Yu Lin, Kuo-Tung Huang, Hsu-Ching Kao, Ying-Tang Fang, Chi-Han Huang, Ya-Ting Chang, Yi-His Wang, Chin-Chou Wang, Meng-Chih Lin

**Affiliations:** 1 Division of Pulmonary and Critical Care Medicine, Department of Internal Medicine, Kaohsiung Chang Gung Memorial Hospital, Chang Gung University College of Medicine, Niaosung, Kaohsiung, Taiwan; 2 Department of Respiratory Therapy, Kaohsiung Chang Gung Memorial Hospital, Chang Gung University College of Medicine, Kaohsiung, Taiwan; 3 Department of Respiratory Care, Chang Gung University of Science and Technology, Chiayi, Taiwan; Azienda Ospedaliero Universitaria Careggi, ITALY

## Abstract

**Background:**

Immunoparalysis was observed in both patients with cancer and sepsis. In cancer patients, Cytotoxic T lymphocyte antigen-4 and programmed cell death protein 1/programmed death-ligand 1 axis are two key components of immunoparalysis. Several emerging therapies against these two axes gained significant clinical benefit. In severe sepsis patients, immunoparalysis was known as compensatory anti-inflammatory response syndrome and this has been suggested as an important cause of death in patients with sepsis. It would be interesting to see if immune status was different in severe sepsis patients with or without active cancer. The aim of this study was to assess the differences in immune profiles, and clinical outcomes between severe sepsis patients with or without cancer admitted to ICU.

**Methods:**

A combined retrospective and prospective observational study from a cohort of adult sepsis patients admitted to three medical ICUs at Kaohsiung Chang Gung Memorial Hospital in Taiwan between August 2013 and June 2016.

**Results:**

Of the 2744 patients admitted to the ICU, 532 patients with sepsis were included. Patients were divided into those with or without active cancer according to their medical history. Of the 532 patients, 95 (17.9%) patients had active cancer, and 437 (82.1%) patients had no active cancer history. Patients with active cancer were younger (p = 0.001) and were less likely to have diabetes mellitus (p < 0.001), hypertension (p < 0.001), coronary artery disease (p = 0.004), chronic obstructive pulmonary disease (p = 0.002) or stroke (p = 0.002) compared to patients without active cancer. Patients with active cancer also exhibited higher baseline lactate levels (p = 0.038), and higher baseline plasma interleukin (IL)-10 levels (p = 0.040), higher trend of granulocyte colony-stimulating factor (G-CSF) (p = 0.004) compared to patients without active cancer. The 14-day, 28-day and 90-day mortality rates were higher for patients with active cancer than those without active cancer (P < 0.001 for all intervals).

**Conclusions:**

Among patients admitted to the ICU with sepsis, those with underling active cancer had higher baseline levels of plasma IL-10, higher trend of G-CSF and higher mortality rate than those without active cancer.

## Background

Cancer is the leading cause of death worldwide and causes heavy socioeconomic impact [1–3]. Among cancer patients, death due to sepsis-related multi-organ failure is more frequent than death due to cancer itself [4–7]. Soares et al. found that cancer patients admitted to intensive care units (ICUs) had an in-hospital mortality rate comparable to ICU patients without cancer [8]. However, debate over cancer patient admission to ICUs has been increasing due to their poor prognosis, increasing demand for home hospice care [9–13]. In fact, many cancer patients return to their daily activities upon recovery from a sepsis episode [4, 5].

Immunoparalysis was observed in both patients with cancer and sepsis [14, 15]. In cancer patients, cytotoxic T lymphocyte antigen-4 and programmed cell death protein 1/programmed death-ligand 1 axis are key components of immunoparalysis [16, 17]. Several emerging therapies against these two axes gained significant clinical benefit [18–21]. In severe sepsis patients, immunoparalysis was known as compensatory anti-inflammatory response syndrome and this has been suggested as an important cause of death in patients with sepsis [22–24]. It would be interesting to see if immune status was different in severe sepsis patients with or without active cancer. In this study, we would like to assess the immune status in severe sepsis patients with or without active cancer and their impact on 14-, 28- and 90-day mortality.

## Methods

### Patient population selection

We conducted a prospective observational study between August 2013 and May 2016 at Kaohsiung Chang Gung Memorial Hospital in Taiwan. Patients with severe sepsis or septic shock who were admitted to medical ICUs were included. All patients were screened for eligibility at the time of admission to the ICU. Patients were excluded if they were younger than 18 years old, receive G-CSF 1 week prior to ICU admission or had an ICU wait time longer than 24 hours after sepsis was diagnosed. This study aimed to analyze baseline and trend of cytokine levels in patients with and without active malignancy, and their impact on 14-, 28- and 90-day mortality. Besides patients with baseline and trend of cytokines available in the prospective part of study, we also retrospectively collected clinical parameters and outcomes of sepsis patients who did not join the study in the same study period. The data were combined for analyzing the differences in clinical parameters between sepsis patients with or without active cancer requiring ICU admission.

### Outcomes

Our primary outcome was the impact of baseline and trend of cytokine levels in sepsis patients with and without active malignancy. Baseline and trend of cytokine levels which were significant difference between patients with or without cancer were used to analyzed their impact on 14-, 28- and 90-day mortality rates. The clinical parameters included age, body mass index (BMI), sex, history of diabetes mellitus (DM), hypertension, coronary artery disease (CAD), chronic obstructive pulmonary disease (COPD), cirrhosis, stroke, chronic kidney disease (CKD), Sequential Organ Failure Assessment (SOFA) score, albumin, C-reactive protein (CRP), lactate, procalcitonin, oxygenation index (OI). Day 1 was defined as the first day of ICU admission. All patients were followed-up until death or until discharge from the hospital. This is a combined retrospective and prospective observational study. The study was approved by the Institutional Review Board of Chang Gung Memorial Hospital with written informed consent obtained from patients or their surrogates in patients agreed cytokine levels (n = 151). The need for informed consent was waived in retrospectively collected clinical parameters and outcomes of sepsis patients who did not join the prospective study in the same study period (n = 381).

### Definitions

We followed the 2001 international guidelines by Surviving Sepsis Campaign [25] for the definition of severe sepsis and septic shock. All patients enrolled in our study before February 2016 were thoroughly evaluated, and all patients met the criteria for sepsis according to the Third International Consensus Definitions for Sepsis and Septic Shock (Sepsis-3) [26]. Subsequently, we adapted to the new definition of sepsis using Sepsis-3 for patient enrollment since 2016 February.

### Plasma preparation and cytokine levels measurement

Whole blood (20 ml) obtained from patients was immediately mixed with heparin tube (BD, Franklin Lakes, NJ, USA). Whole blood was centrifuged at 400*g* for 30 minutes to separate the plasma from whole blood and was stored at -80°C. MILLIPLEX^®^ MAP kits (Human Cytokine/Chemokine Magnetic Bead Panel, HCYTOMAG-60K, EMD Millipore, Darmstadt, Germany) were used to quantify the following plasma cytokine levels: IL-6, IL-10, granulocyte colony-stimulating factor (G-CSF), tumor necrosis factor-α (TNF-α), and human leukocyte antigen D—related (HLA-DR). The MAGPIX System device (Millipore, Darmstadt, Germany) was used to analyze standards and samples by using a 5-parameter logistic curve fitting model (5PL) by the MILLIPLEX^®^ Analyst 5.1 software (Millipore, Darmstadt, Germany).

### Statistical analyses

Statistical analyses were performed using MedCalc (version 14.10.2). A receiver operating characteristic (ROC) curve was used to determine the best cut-off values of the prognostic factors which were statistically significant in univariable analysis. Categorical variables were compared using the chi-square test or Fisher’s exact test where appropriate, and continuous variables were analyzed using Student’s t-test or the Mann—Whitney U test where appropriate. A p-value < 0.05 was considered statistically significant.

## Results

### Patient characteristics

**Fig 1:**
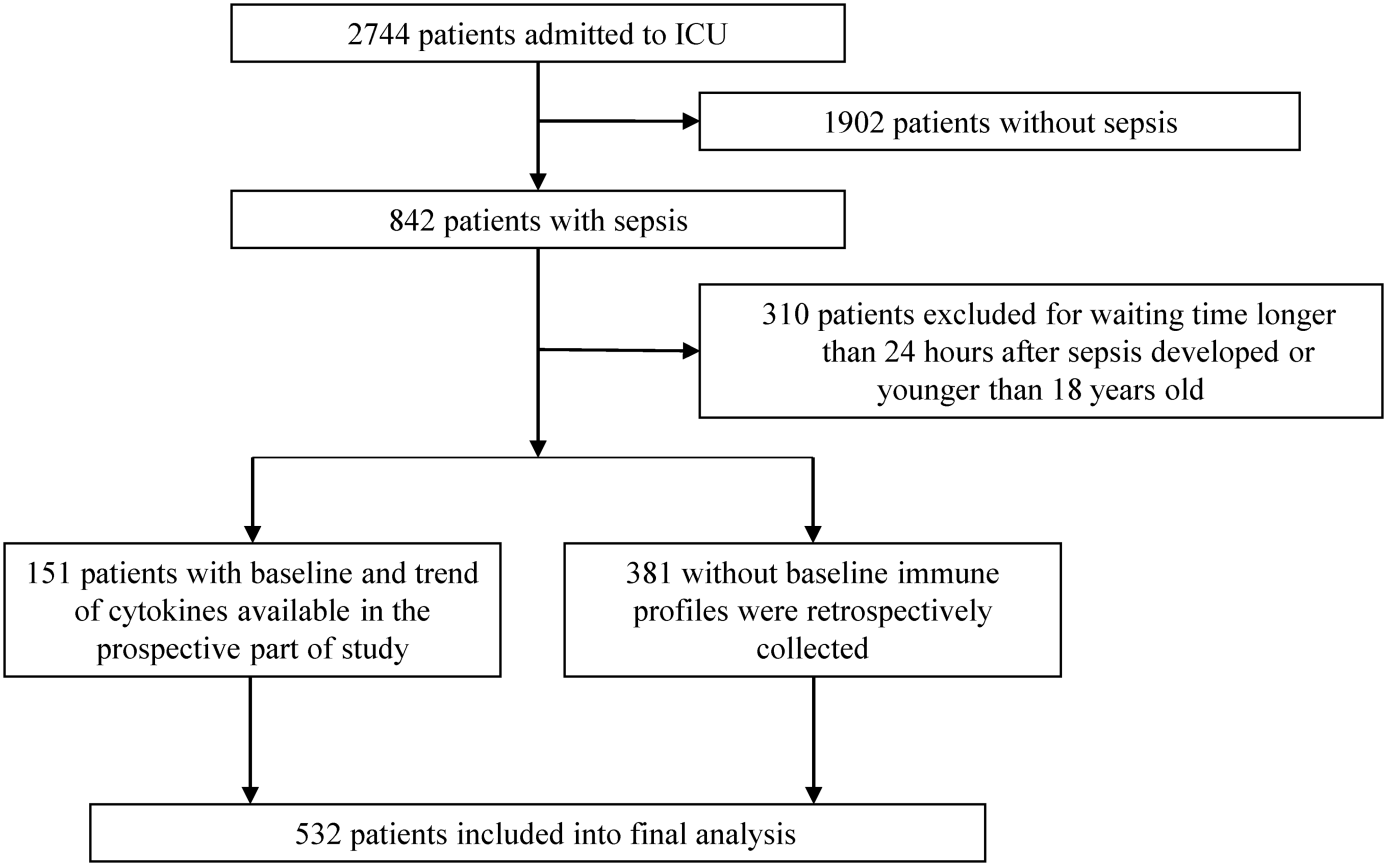
Patient inclusion and assignment.

Of the 2744 patients admitted to the ICU of Kaohsiung Chang Gung Memorial Hospital between August 2013 and June 2016, 532 sepsis patients were included into final analyses (Fig 1). The mean age of participants was 66.5±15.2 years old (range from 21–97 years old) Of the 532 patients, 122 patients (22.9%) had cancer history and 410 (82.1%) patients had no cancer history. Of the 122 patients with cancer history, 27 (22.1%) patients were diagnosed with early-stage cancer post—complete resection without evidence of cancer recurrence. These patients were classified as the inactive cancer group. Ninety-five of the 122 patients (77.9%) had cancers that were either inoperable or recurred after surgical resection. These patients were classified as the active cancer group. The leading active cancer in our ICU was lung cancer (n = 19, 20%), followed by head and neck cancer (n = 17, 17.9%) and hematologic malignancies (n = 12, 13.7%).

### Baseline clinical parameters of sepsis patients and their impact on prognosis

Patients with active cancer were younger (p = 0.001) and exhibited lower rates of DM (27.4% vs. 47.6%, p < 0.001), hypertension (32.6% vs. 56.8%, p < 0.001), CAD (13.7% vs. 27.5%, p = 0.004), COPD (3.2% vs. 13.5%, p = 0.002) and stroke (9.5% vs. 23.1%, p = 0.002) compared to patients without active cancer (Table 1). Patients with active cancer exhibited higher baseline lactate levels (p = 0.038) than patients without active cancer (Table 1). There was no significant difference between patients with and without active malignancy regarding BMI, sex, history of cirrhosis, CKD, SOFA score, albumin, CRP, procalcitonin and OI.

**Table 1:** Baseline clinical parameters between ICU sepsis patients with or without underling active malignancy.

Clinical parameters	ALL patients (n = 532)	With *active malignancy* (n = 95, 17.9%)	Without *active malignancy* (n = 437, 82.1%)	p
Age, years	66.5(15.2)	62.2(12.8)	67.4(15.5)	0.001
BMI	22.8(5.0)	22.0(4.3)	23.0(5.1)	0.090
Sex				0.300
Male	314 (59.0)	61(64.2)	253(57.9)	
Female	218 (41.0)	34(35.8)	184(42.1)	
Diabetes mellitus	234 (44.0)	26 (27.4)	208 (47.6)	<0.001
Hypertension	279 (52.4)	31 (32.6)	248 (56.8)	<0.001
CAD	133 (25.0)	13 (13.7)	120 (27.5)	0.004
COPD	62 (11.7)	3 (3.2)	59 (13.5)	0.002
Cirrhosis	43 (8.1)	10 (10.5)	33 (7.6)	0.404
Stroke	110 (20.7)	9 (9.5)	101 (23.1)	0.002
CKD	141 (26.5)	20 (21.1)	121 (27.7)	0.201
APACHE II score	25.0 (8.8)	23.8 (8.3)	25.2 (8.9)	0.180
*SOFA score*	9.4(3.9)	9.4 (3.9)	9.5 (3.5)	0.770
Albumin (g/dl)	2.8(1.2)	2.7 (0.5)	2.8 (1.4)	0.675
CRP (mg/L)	156.0(116.5)	165.5 (133.7)	154.1 (113.1)	0.454
Lactate (mg/dL)	33.9(30.7)	42.0 (36.5)	32.2 (29.2)	0.038
Procalcitonin (ng/ml)	25.4(49.0)	27.8 (54.7)	24.8 (47.7)	0.682
OI (cmH2O/mmHg)	9.6(9.7)	10.3 (10.1)	9.4 (9.7)	0.392

Abbreviations: BMI, body mass index; CKD, Chronic kidney disease; COPD, Chronic obstructive pulmonary disease; CAD, Coronary artery disease; CRP, C-reactive Protein; SOFA, Sequential Organ Failure Assessment score

**Fig 2:**
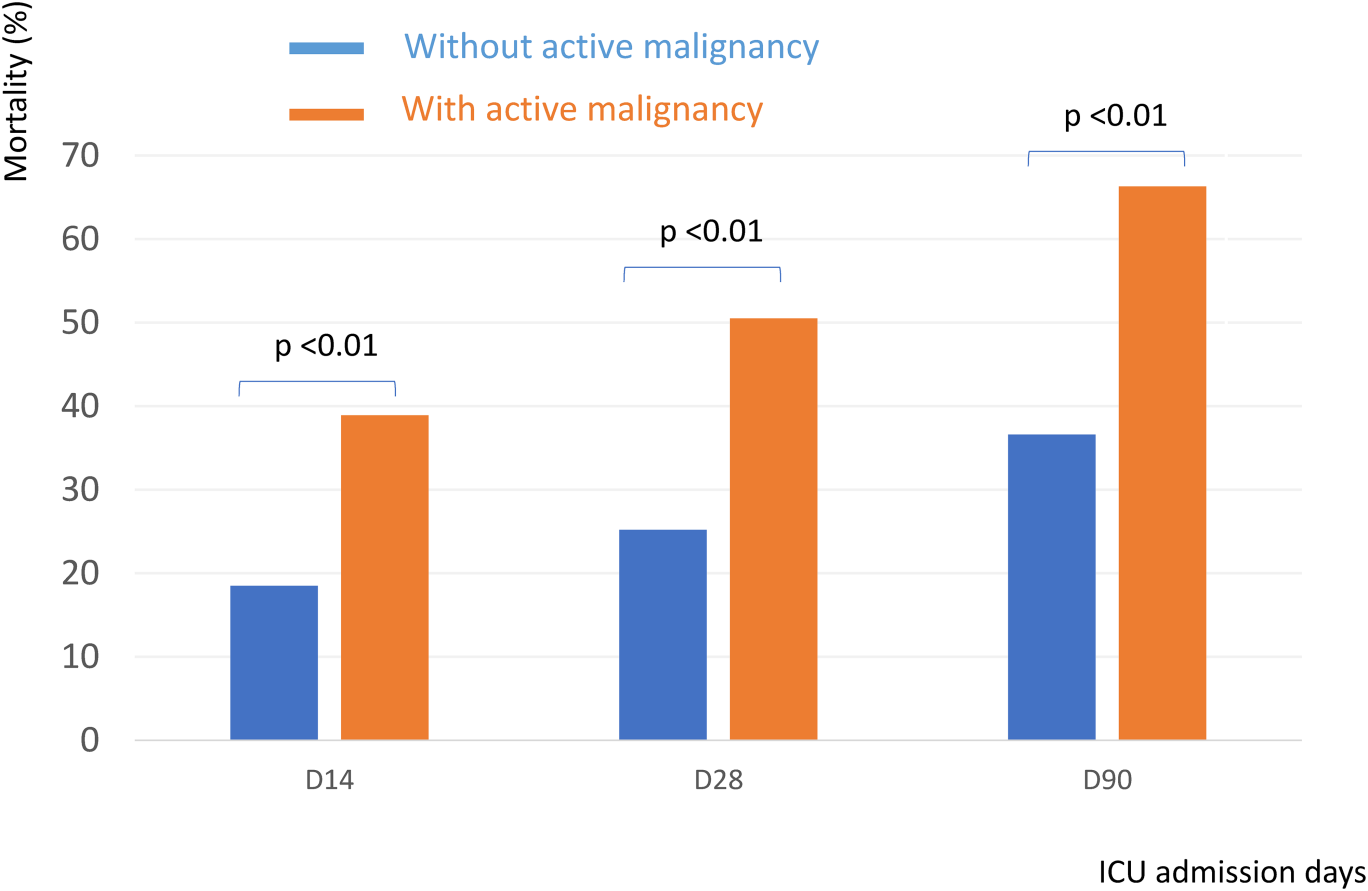
ICU mortality in patients with or without active malignancy.

**Fig 3:**
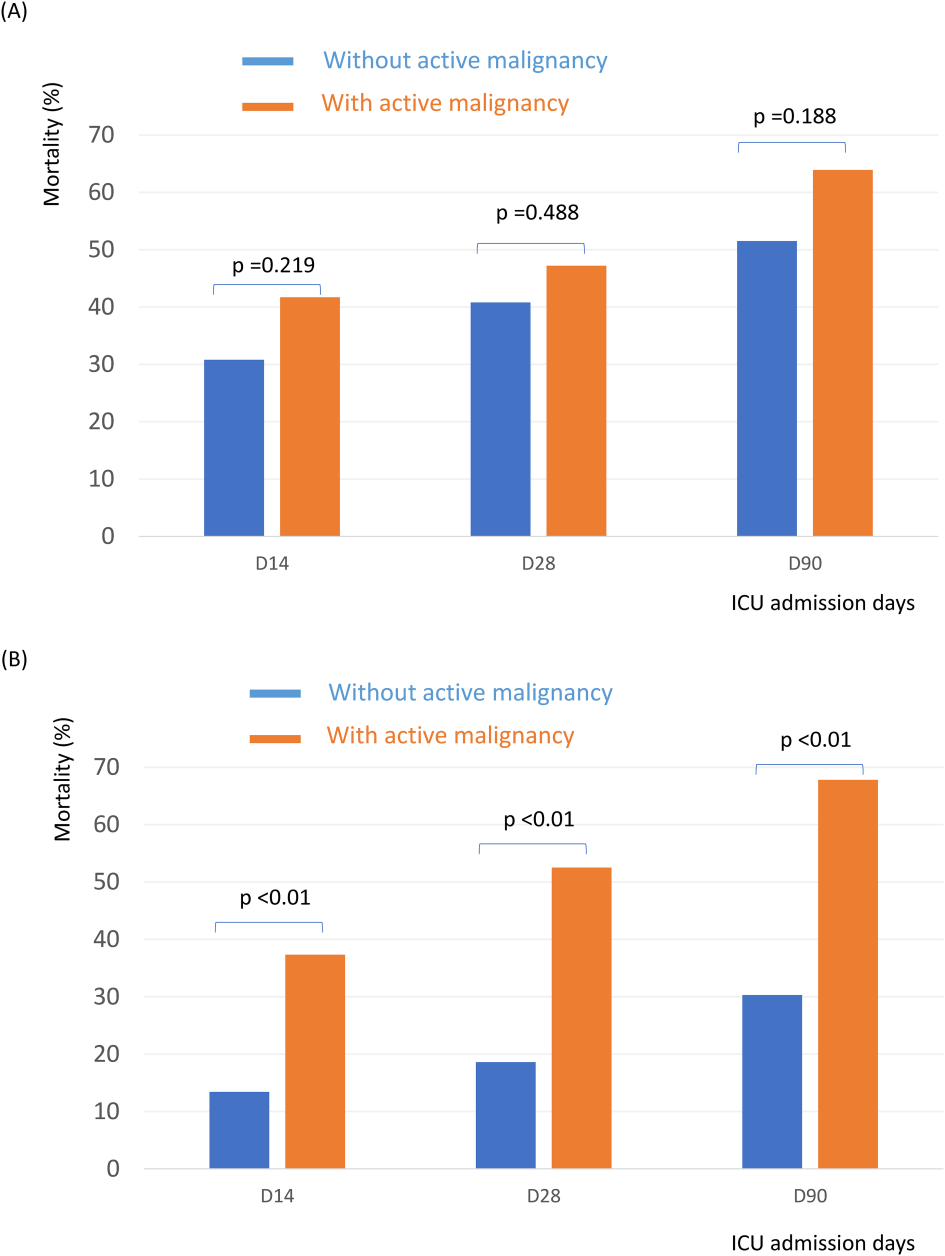
ICU mortality in septic shock patients with or without active malignancy (3A); ICU mortality in patients without shock with or without active malignancy (3B).

Patients with active cancer had higher 14-day (38.9% vs. 18.5%, P < 0.001), 28-day (50.5% vs. 25.2%, P < 0.001) and 90-day (66.3% vs. 36.6%, P < 0.001) mortality rates than patients without active cancer (Fig 2). Of the 532 patients, 166 patients had septic shock. In subgroup of patients with septic shock, those who had active cancer had equivalent mortality rate than those without cancer. (Fig 3A) However, in patients without shock, those who had active cancer had higher mortality rate than those without (Fig 3B).

### Baseline Immune profiles of sepsis patients and their impact on prognosis

**Fig 4:**
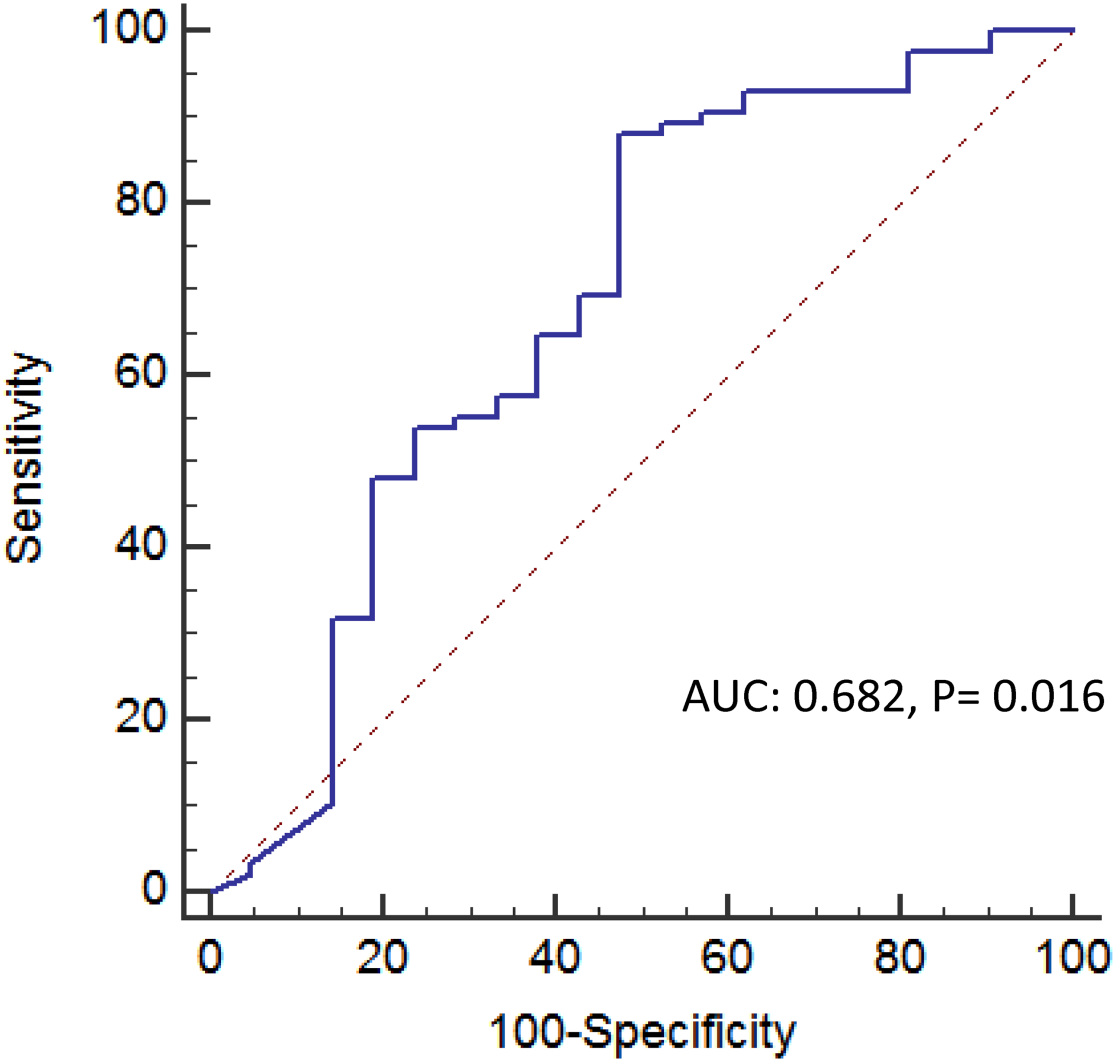
The ROC curve of IL-10 for 28-day mortality prediction.

**Fig 5:**
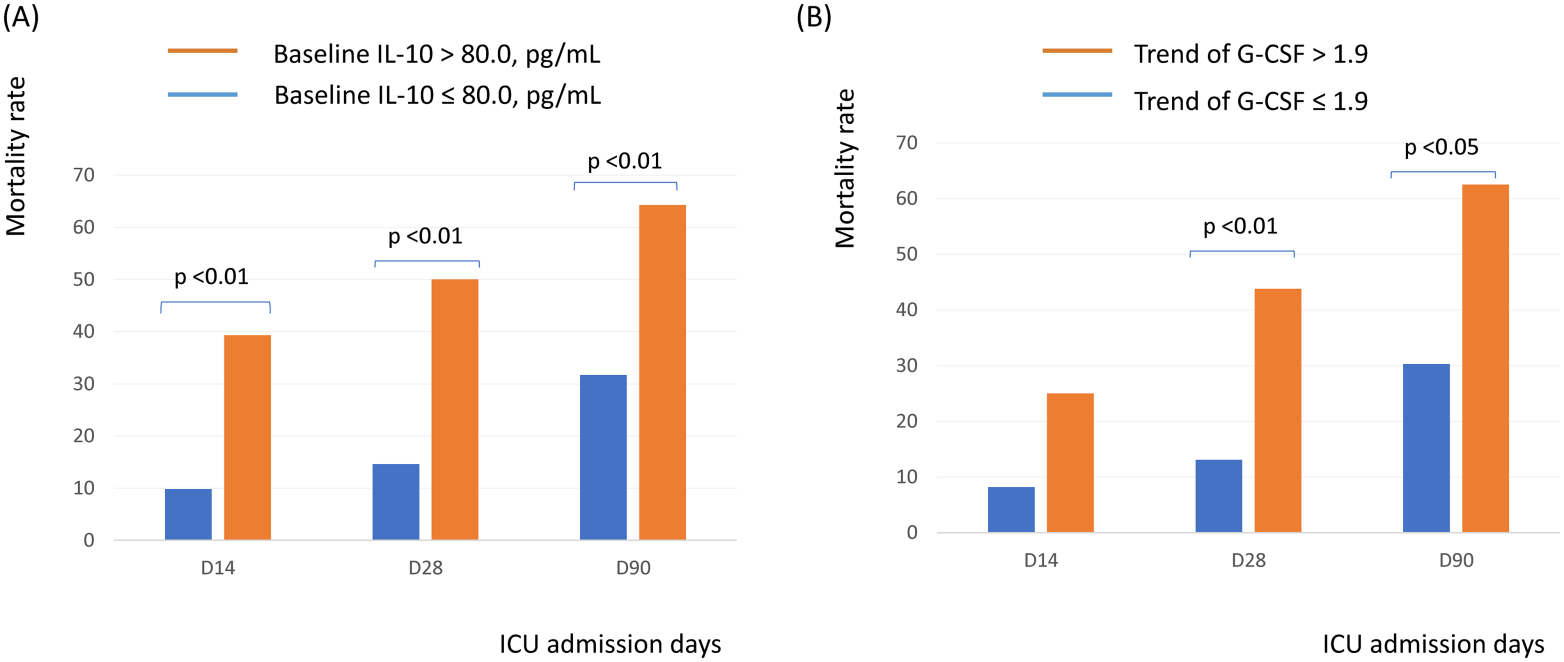
Influence of (A) baseline IL-10 and (B) trend of G-CSG and clinical outcome.

Of the 532 patients, day 1 cytokine levels were available in 151 patients. Patients with active cancer had higher baseline IL-10 levels than those without cancer (p = 0.023) (Table 2). There was no significant difference between patients with and without active malignancy regarding baseline IL-6, G-CSF and TNF-α levels. The optimal cut-off point of IL-10 for all patients determined by the ROC curve and Youden’s Index was 80 pg/ul. (Fig 4) Patients were divided into high or low levels of IL-10 based on this cut-off value. Patients with high IL-10 levels had higher 14-day (p < 0.001), 28-day (p < 0.001) and 90-day (p = 0.002) mortality rates than patients with low IL-10 levels (Fig 5A).

**Table 2:** 

Immune profiles (Median)	ALL patients (n = 95)	With *active malignancy (n = 19)*	Without *active malignancy (n = 76)*	p
IL-6				
D1 (pg/uL)	52.0 (96.7)	85.1 (291.4)	51.3 (93.5)	0.302
D3/D1 (%)	0.6 (0.8)	0.8 (0.9)	0.6 (0.7)	0.129
IL-10				
D1 (pg/uL)	16.7 (56.3)	34.0 (68.0)	15.1 (52.9)	0.040
D3/D1 (%)	0.9 (0.6)	0.8 (1.1)	0.9 (0.6)	0.649
G-CSF				
D1 (pg/uL)	70.9 (117.9)	89.5 (357.4)	70.8 (103.2)	0.827
D3/D1 (%)	0.6 (0.8)	1.5 (1.9)	0.6 (0.7)	0.004
TNF-α				
D1 (pg/uL)	33.5 (45.2)	44.6 (87.0)	32.6 (40.8)	0.220
D3/D1 (%)	0.9 (0.5)	0.9 (0.4)	0.9 (0.5)	0.405
HLA-DR				
D1 (%)	92.3 (16.0)	91.6 (11.6)	92.6 (17.2)	0.487
D3/D1 (%)	1.0 (0.1)	0.9 (0.1)	1.1 (0.1)	0.508

Abbreviations: G-CSF, granulocyte colony-stimulating factor; HLA-DR: human leukocyte antigen D—related; IL, interleukin; TNF: tumor necrosis factor.

### Trend of Immune profiles of sepsis patients and their impact on prognosis

Of the 151 patients having day 1 cytokine levels and HLA-DR expression, day 3 cytokine levels and HLA-DR expression were available in 138 and 133 patients, respectively. Patients with active cancer had higher trend of G-CSF (p = 0.004) than those without cancer (Table 2). There was no significant difference between patients with and without active malignancy regarding trend of IL-6, IL-10, TNF-α levels and HLA-DR expression. The optimal cut-off point of trend of G-CSF determined by the ROC curve and Youden’s Index was 1.9. Patients were divided into high or low trend of G-CSF based on this cut-off value. Patients with high trend of G-CSF levels had higher 28-day (p = 0.002) and 90-day (p = 0.011) mortality rates than patients with low tend of G-CSF levels (Fig 5B).

## Discussion

In our cohort, sepsis patients with underlying active malignancy accounted for 17.9% of all ICU admissions, similar to a previous study with a range of 15–20% [8, 27]. Our study revealed that sepsis patients with active cancer, when compared to those without active cancer, were predominantly younger in age and were less likely to have chronic illnesses such as diabetes mellitus, hypertension, coronary artery disease, chronic obstructive pulmonary disease and stroke. Although older patients have a higher incidence and a higher prevalence of malignancy in general [28–32], they are also more likely to receive home hospice care compared to younger cancer patients in Taiwan [33]. We presumed this difference made our cancer patients admitted to ICU younger and had less chronic comorbidities.

Our study revealed that patients with active cancer had higher baseline plasma IL-10 levels and patients with higher baseline IL-10 had higher 14-, 28- and 90-day mortality rates.

Previous studies found higher IL-10 level associated with immunoparalysis and poor outcome in patients with septic shock [34]. A statistically non-significant increasing of IL-10 in patients with neutropenia was noted in our study. (IL-10 in neutropenic vs. non-neutropenic patients: 308.7 vs. 119.9 pg/ul, p = 0.079). In a study by Matti et al., IL-10 levels were noted to be an early predictor of gram-negative bacteremia in febrile neutropenic patients [35]. Vincas et al. also notes elevated IL-10 levels in febrile, neutropenic, pediatric patients with cancer [36]. Additionally, Sachin et al. noted elevated IL-10 levels in patients with pneumonia, which was shown to be associated with one-year all-cause mortality [37]. While it is probable that elevated IL-10 levels are related to poor prognosis in cancer patients admitted to the ICU, further studies need to be done in this area. IL-6, a pro-inflammatory cytokine [38], is associated with suppression of prostate cancer metastases [39] and is higher in patients with lung cancer [40]. No difference was noted in plasma IL-6 levels among sepsis patients with or without active cancer in our study.

G-CSF is essential in the production of neutrophils during infection, and is responsible for restoration of polymorphonuclear cell function in cancer patients [41]. Reilly et al. found higher baseline G-CSF level in neutropenic patients [42]. However, a statistically non-significant lower trend of G-CSF in patients with neutropenia was noted in our study. (G-CSF trend in neutropenic vs. non-neutropenic patients: 3.2 vs. 5.5, p = 0.780). No significant difference was noted in baseline plasma G-CSF levels among sepsis patients with and without active cancer in our study. However, patients with active cancer had higher trend of plasma G-CSF levels and patients with higher trend of plasma G-CSF levels had higher 28- and 90-day mortality rates. These correlations were seldom mentioned in previous study and need further study to validate it. Serum and plasma TNF-α levels have been shown to increase significantly among patients with sepsis, particularly in culture-positive patients [43, 44]. One study showed that anti-TNF-α therapy increased the risk of non-Hodgkin’s lymphoma [45]. Our study revealed no significant difference in plasma TNF-α levels among sepsis patients with and without active cancer. Decreased monocyte HLA-DR expression during protracted sepsis measured by flow cytometry is a marker of immune paralysis in critically septic patients [46]. Patients with lower monocyte HLA-DR expression increased risk of bacterial sepsis after liver transplantation [47]. Higher HLA-DR expression in cervical adenocarcinoma patient was found associated with longer disease-free survival and disease-specific survival [48]. Our study revealed no significant difference in monocyte HLA-DR expression among sepsis patients with and without active cancer.

A study by Soares et al. showed that cancer patients admitted to ICU had 30% overall hospital mortality rate which was equivalent to that of patients without malignancy [8]. However, our study revealed cancer patients with sepsis requiring ICU admission had a dismal prognosis with a 28-day mortality rate up to 50.5%. The differences in mortality outcomes between our study and the study by Soares et al. may be related to different patient inclusion criteria. First, the previous study included 66% patients with locoregional cancer, which was only 21.0% in our study. Second, the previous study included only 15% patients with sepsis, while we only included patients with sepsis. Third, the previous study included only 27% patients requiring ventilator support; however, 91.8% of our patients required ventilator support. Finally, cancer patients in our study had higher baseline lactate levels than those in the previous study. Higher lactate levels was found to be poor prognostic factor in the previous studies [49].

Our study has several limitations. First, day 1 and 3 circulating cytokines levels were only available in 151 and 138 patients. Further studies are required to elucidate the true negative rate and the statistical power of this study to show significant differences in plasma IL-6, TNF-α and monocyte HLA-DR expression between patients with and without cancer. Second, detailed treatment modalities of patients prior to ICU admission were not available. We are uncertain how many patients, if any, received target therapies, anti-angiogenesis agents, or immunotherapy prior to ICU admission. Whether or not these therapies affected patient immune parameters and subsequent clinical outcomes needs to be further explored.

## Conclusions

Sepsis patients with underling active malignancy requiring ICU admission had distinct immune profiles and worse outcomes than those without active malignancy.

## Supporting information

S1 File Raw data. (MC1)
